# 1*s*-intraexcitonic dynamics in monolayer MoS_2_ probed by ultrafast mid-infrared spectroscopy

**DOI:** 10.1038/ncomms10768

**Published:** 2016-02-25

**Authors:** Soonyoung Cha, Ji Ho Sung, Sangwan Sim, Jun Park, Hoseok Heo, Moon-Ho Jo, Hyunyong Choi

**Affiliations:** 1 School of Electrical and Electronic Engineering, Yonsei University, Seoul 120-749, Korea; 2 Center for Artificial Low Dimensional Electronic Systems, Institute for Basic Science (IBS), Pohang University of Science and Technology (POSTECH), Pohang 790-784, Korea; 3 Division of Advanced Materials Science, Pohang University of Science and Technology (POSTECH), Pohang 790-784, Korea

## Abstract

The 1*s* exciton—the ground state of a bound electron-hole pair—is central to understanding the photoresponse of monolayer transition metal dichalcogenides. Above the 1*s* exciton, recent visible and near-infrared investigations have revealed that the excited excitons are much richer, exhibiting a series of Rydberg-like states. A natural question is then how the internal excitonic transitions are interrelated on photoexcitation. Accessing these intraexcitonic transitions, however, demands a fundamentally different experimental tool capable of probing optical transitions from 1*s* ‘bright' to *np* ‘dark' states. Here we employ ultrafast mid-infrared spectroscopy to explore the 1*s* intraexcitonic transitions in monolayer MoS_2_. We observed twofold 1*s*→3*p* intraexcitonic transitions within the A and B excitons and 1*s*→2*p* transition between the A and B excitons. Our results revealed that it takes about 0.7 ps for the 1*s* A exciton to reach quasi-equilibrium; a characteristic time that is associated with a rapid population transfer from the 1*s* B exciton, providing rich characteristics of many-body exciton dynamics in two-dimensional materials.

## Abstract (web summary)

Excitons—bound electron-hole pairs—in two-dimensional transition-metal dichalcogenides can exhibit a rich spectrum of excited states. Here, the authors use ultrafast mid-infrared spectroscopy to explore the dynamics of these so-called 1s-intraexcitonic transitions in monolayer molybdenum disulphide.

## Graphical abstract

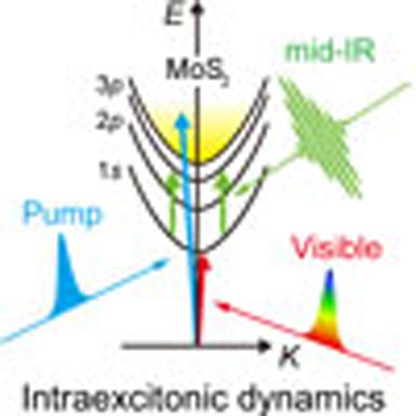

Photogenerated electron-hole (e–h) pairs in solids create bound states, whose elementary quasiparticle state is called 1*s* exciton in a Wannier–Mott exciton model. Since the optoelectronic response is governed by the light-induced dynamic behaviour of this elementary ground state, knowledge of the 1*s* exciton response to the optical stimuli has been a crucial issue in many optoelectronic applications, such as phototransistors^1^,^2^, photovoltaics^3^, light-emitting diodes^4^,^5^, van der Waals heterostructure-based optoelectronics^6^,^7^,^8^ and valleytronic device applications^5^,^9^,^10^,^11^,^12^. In transition metal dichalcogenides (TMDCs), this is particularly the case as the two-dimensional (2D) materials approach a monolayer limit, where the reduced dielectric screening results in a strong Coulomb interaction^13^,^14^,^15^,^16^,^17^,^18^,^19^,^20^,^21^, leading to an unusually large 1*s* exciton binding energy *E*_bind_, typically a few hundreds of meV below the electronic bandgap of a few eV (refs 16, 22, 23, 24, 25, 26, 27).

Above the fundamental 1*s* exciton, theories predicted the presence of densely spaced exciton states in monolayer MoS_2_ with 1*s* exciton *E*_bind_ of 0.4–0.54 eV (refs 18, 19, 20, 21, 28, 29, 30, 31), whose (*s*-like) bright and (*p*-like) dark exciton characters were later confirmed by a series of seminal experiments via linear one-photon absorption^21^,^22^, two-photon photoluminescence excitation (PLE)^22^,^23^,^24^,^32^ and nonlinear wave-mixing spectroscopy^25^,^32^, whereby *E*_bind_ was experimentally measured to be between 0.22 (ref. 26) and 0.44 eV (refs 23, 26); the reported *E*_bind_, however, shows somewhat discrepancy depending on the measurement methods and is varied from samples to samples^23^,^26^,^27^. These experimental techniques, although they are appropriate to clarify the optical state of the excitons, may address indirectly the dynamic transient information between the 1*s* ‘bright' and the excited *np* ‘dark' exciton (*n* is the principle quantum number); we denoted the exciton states in analogy to the hydrogen series^21^. By contrast, if one measures the 1*s*→*np* transitions, then the data should describe the internal excitonic transients, directly providing the transient optical nature of the fundamental 1*s* exciton dynamics. This, so called intraexcitonic spectroscopy^33^, fundamentally differs from band-to-band and other time-resolved spectroscopies^8^,^34^,^35^,^36^, and the technique can not only explain the transient response of the 1*s* exciton, but more importantly, may provide experimental manoeuvre in exploiting the photoinduced excitonic responses to the TMDC-based optoelectronic devices. For example, knowledge of the 1*s* and n*p* exciton energies and their associated dynamics afford the first-order quantitative information on the exciton dissociation energy, where in an ideal case at least *E*_bind_/e (e is the electron charge) of an external or internal potential is required to dissociate the bound e–h pairs. In addition, because intraexcitonic spectroscopy can access the *p*-like dark excitons, one may design a scheme of coupling an infrared (IR) light to the 2D TMDC materials, via below-gap two-photon excitation, for the light-harnessing applications.

Here we explore the 1*s* intraexcitonic transient dynamics in monolayer MoS_2_ by using time-resolved mid-IR spectroscopy. Inspired by a theoretical GW–Bethe–Salpeter result^19^, where the fundamental 1*s*→2*p* transitions are predicted to be 0.32 and 0.3 eV for the A and B exciton in isolated, suspended monolayer MoS_2_, we employed an ultrafast mid-IR spectroscopy (0.23–0.37 eV probe) in conjunction with an ultrafast white-light continuum spectroscopy (Fig. 1a). The mid-IR measurements show that there are two 1*s*→3*p* transitions for A and B exciton and 1*s*→2*p* between 1*s* A and 2*p* B exciton. The time-dependent IR absorption rapidly subdues over broad probe–photon energies, representing the transient absorption from the 1*s* to the quasi-continuum states after pump excitation.

## Results

### Time-resolved intraexcitonic and band-to-band dynamics

**Figure 1:**
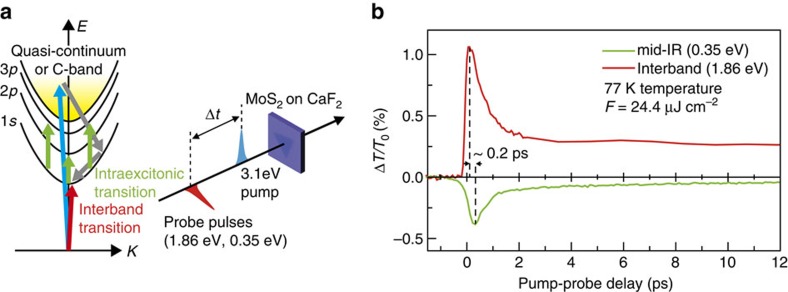
Ultrafast intraexcitonic and band-to-band spectroscopy in monolayer MoS_2_. (**a**) Schematic illustration of the ultrafast intraexcitonic (green) and conventional band-to-band (red) interband spectroscopy. The 3.1 eV optical pump (blue arrow) creates e–h pairs from ground to quasi-continuum states or C-band. The mid-IR pulse (green) measures the 1*s*→*np* transitions, while the white-light continuum pulse measures the ground-to-1*s* transition. (**b**) Transient dynamics of Δ*T*/*T*_0_ measured at two representative energies of 1.86 eV (red) and 0.35 eV (green). The positive sign of Δ*T*/*T*_0_ is observed for 1.86 eV probe, while 0.35 eV probe exhibits a negative sign. A clear temporal delay (∼0.2 ps) between the two rising transients was observed. The experimentally determined temporal error bar for each time-zero (20 fs for 0.35 eV and 8 fs for 1.86 eV) is discussed in Supplementary Fig. 8.

**Figure 2:**
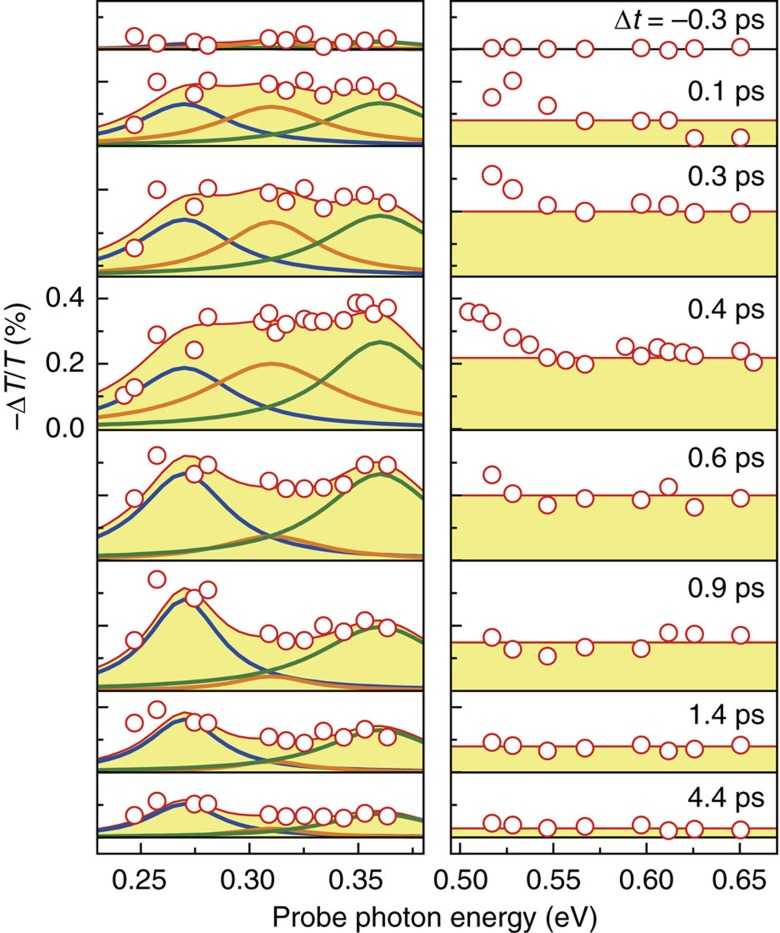
Temporally and spectrally resolved transitions from 1s state. Left panel is the mid-IR transient and right panel is the IR transient dynamics. The transient spectra (red dots) are fitted by three Lorentzian oscillators (blue, orange and green) for the mid-IR range and a step function (red) for the IR range. The red solid in left panel is the sum of three intraexcitonic oscillators. We note that it was not possible to fit all the mid-IR spectra if only two oscillators were used.

**Figure 3:**
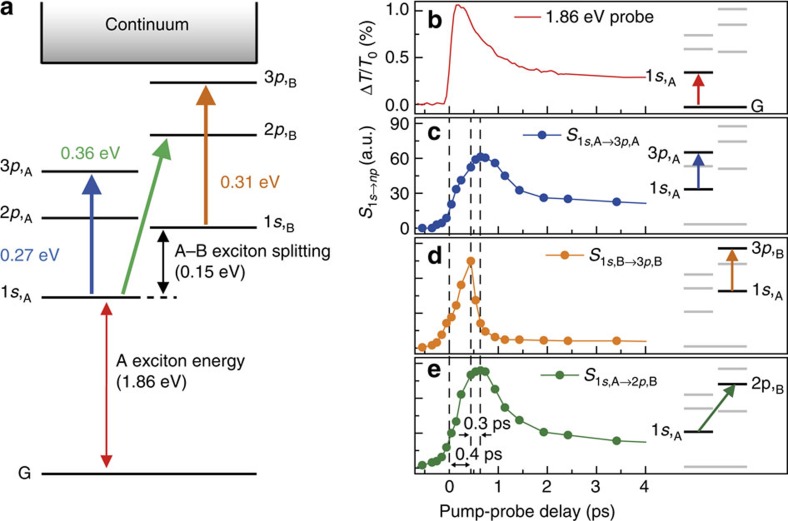
Schematic for 1*s* intraexcitonic transition and relevant spectral weight. (**a**) Energy diagram of the ground state (G), and the fundamental excitons (1*s*_,A_ and 1*s*_,B_) and the higher excited *np* dark excitons in shown. Transition energies of three oscillators are indicated by blue (0.27 eV), orange (0.31 eV) and green (0.36 eV) arrows. The transient band-to-band dynamics (**b**) is directly compared with the intraexcitonic absorption dynamics (**c**–**e**). Transient dynamics of the intraexcitonic spectral weight parameter *S*_1s→*np*_ for each three oscillator are shown at each row: (**c**) 1*s*_,A_→3*p*_,A_, (**d**) 1*s*_,B_→3*p*_,B_ and (**e**) 1*s*_,A_→2*p*_,B_, respectively. Dashed lines show the maximum *S*_1s→*np*_ peak for each intraexcitonic transition.

**Figure 4:**
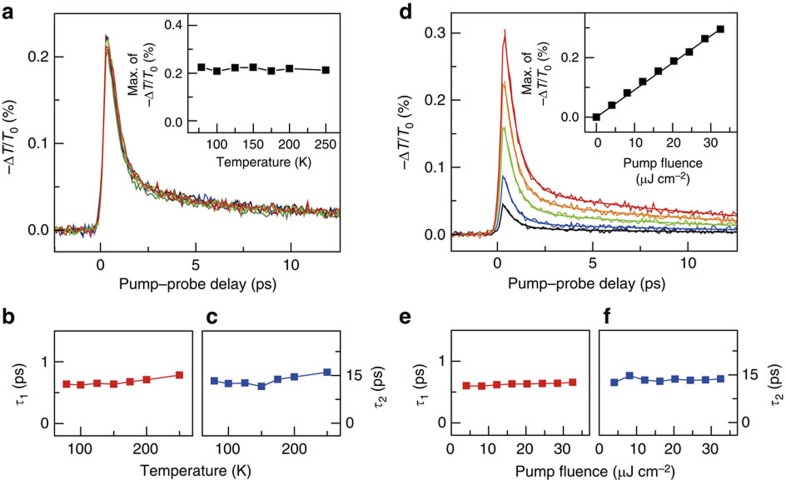
Temperature- and fluence-dependent mid-IR dynamics. (**a**) Temperature-dependent −Δ*T*/*T*_0_ dynamics measured at 0.6 eV. Inset: the peak value of −Δ*T*/*T*_0_ is plotted as a function of the temperature. No temperature-dependent dynamics were observed, thereby the free-carrier absorption can be excluded in the analysis of Figs 1, 2, 3. We performed fitting using a biexponential function. The summarized results are shown in **b** for the fast *τ*_1_ and in **c** for the slow decay component *τ*_2_, where both components are temperature-independent. (**d**) Fluence-dependent −Δ*T*/*T*_0_ dynamics measured at 0.6 eV probe. Inset: the peak −Δ*T*/*T*_0_ shows a linear fluence dependence, such that no higher-order nonlinear exciton dynamics were observed. Fluence-dependent fast *τ*_1_ (**e**) and slow decay component *τ*_2_ (**f**). Solid line for each trace is the corresponding biexponential fit. Both *τ*_1_ and *τ*_2_ are independent of the pump fluence, implying that no absorption occurs from the defect states.

The samples used in our experiment were monolayer MoS_2_, grown by chemical vapour deposition method, and were transferred to a CaF_2_ substrate (see Supplementary Note 1 for the sample characterization). As schematically shown in Fig. 1a, the sample was non-resonantly excited by a 70 fs, 3.1 eV pump pulse, and the corresponding differential-transmission changes Δ*T*/*T*_0_ were measured in a vacuum cryostat (Methods). The 3.1 eV pump excites carriers into the quasi-continuum of the A and B excitons^37^,^38^ or into the band-nesting C-band near the Γ point^39^,^40^. The former generates the unbound e–h plasma above the A and B excitons and the latter case experiences a rapid inter-valley scattering into K and K′ valley. Nevertheless, both cases generate carriers in much higher energy compared with the A or B exciton resonance. Figure 1b shows a direct comparison of two representative data measured by mid-IR probe (0.35 eV) and interband A-exciton probe (1.86 eV) with the same pump fluence *F*=24.4 μJ cm^−2^ (equivalent to e–h pair density of 7.4 × 10^12^ cm^−2^ given 15% absorption)^8^,^41^ measured at 77 K. The polarization of pump and probe beam are linear and orthogonal with respect to each other, such that we do not account for the recently discovered valley-exciton-locked selection rule^32^. The fact that two Δ*T*/*T*_0_ transients exhibit an opposite sign implies the kinetic origin of the photoresponses is indeed different. For the 1.86 eV dynamics, the increased probe transmission is typically attributed to the ground-state bleaching^35^,^42^,^43^, where the increased occupation probability of electrons in conduction band and holes in the valance band leads to the reduced probe absorption, that is, increased Δ*T*/*T*_0_>0. On the other hand, given that the 0.35 eV probe is far below the band-to-band A-exciton energy, one may attribute the decreased Δ*T*/*T*_0_<0 (increased probe absorption) to the transition within the bands, that is, intraband free-carrier absorption. However, considering that the mid-IR peak signal is only 36.2% of the 1.86 eV one, we can exclude this possibility because the intraband oscillator strength is much smaller than the interband one, usually by an order of magnitude, as revealed by prior works on the quasi-2D quantum wells^44^ or recent 2D MoS_2_ (ref. 45); as discussed later in Figs 2, 3, 4, we provide compelling experimental evidences to support our rationale (see also Supplementary Note 2 (refs 46, 47, 48)). The increased probe absorption of the mid-IR suggests that there exists an occupied state below the electronic gap.

We find that there exists a clear time departure between the two rising dynamics, where the onset of the 0.35 eV probe peak appears ∼0.2 ps later than the 1.86 eV probe (dashed line in Fig. 1b). Immediately after the pump, the 1.86 eV probe rapidly increases, while the 0.35 eV dynamics emerge rather slowly. We observed that this rapid upsurge of the 1.86 eV is not a local spectral behaviour, but being presented in a broad range of high-energy probes (Supplementary Notes 3 and 4), evidencing the quasi-instantaneous ground-state bleaching^35^. Understanding this high-energy dynamics has been a subject to debate; different investigations have proposed different kinetic origins of the 1*s* exciton, such as exciton linewidth broadening^35^, stimulated emission^42^, dynamic bandgap renormalization^49^ and biexciton formation^38^. As discussed, more details in Supplementary Note 4, both earlier^50^,^51^,^52^ and recent studies^35^,^42^,^53^, have shown that the 3.1 eV photoexcitation into the quasi-continuum of unbound states generates a significant amount of free-carriers. Because the exciton formation occurs after exciton–free carrier scattering, the initial decaying kinetics of mid-IR is slightly delayed compared with the rising transient of the interband one, explaining the observed time-delay between the two transients of Fig. 1b. Since the mid-IR probe can resonantly measure the internal exciton dynamics, the measured intraexcitonic transients are expected to provide pure population dynamics of the ground 1*s* exciton^33^,^54^.

### Temperally and spectrally resolved intraexcitonic dynamics

Figure 2 is the temporally and spectrally resolved mid-IR dynamics. Here we probed not only the broad mid-IR transients (0.23–0.37 eV), but also measured the IR dynamics (0.47–0.67 eV). This scheme affords a simultaneous access to the dynamic transitions from the 1*s* ground exciton to the higher lying *np* excitons or quasi-continuum states. The measured −Δ*T*/*T*_0_ spectra show peculiar energy-dependent behaviours. At Δ*t*≤0.4 ps, the −Δ*T*/*T*_0_ spectra are strongly reshaped, exhibiting a relatively small increase of differential absorption (not absolute absorption) near 0.27 eV compared with the increased absorption around 0.3–0.5 eV. The increased absorption is more prominent at Δ*t*>0.4 ps, where one can see that −Δ*T*/*T*_0_ is gradually larger near 0.27 eV with increasing Δ*t*, and the differential absorption at 0.3–0.5 eV is concurrently smaller with increasing Δ*t*. Between 0.4<Δ*t*≤0.9 ps, −Δ*T*/*T*_0_ above 0.3 eV is rapidly vanished, while the absorption resonance below 0.3 eV is accordingly increased. After Δ*t*>0.9 ps, the absorption resonance below 0.3 eV keeps reserved and it slowly subdues with featureless IR spectra above 0.3 eV.

For a quantitative analysis of the observed transient spectra, we use the following model consisted of multi-oscillator components^55^:


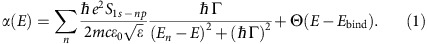


The term of summation represents the intraexcitonic absorption from 1*s* to either A or B excitonic *np* state and the second term Θ(*E*−*E*_bind_) is a step-like transition^21^ from 1*s* to the continuum with *E*_bind_ of 0.44 eV. In the equation, *ɛ* (=4.2) (refs 14, 27) and *ɛ*_0_ are the dielectric constant of monolayer MoS_2_ and the vacuum dielectric constant, respectively. There, the absorption amplitude *S*_1*s*→*np*_, or the spectral weight of the intraexcitonic 1*s*→*np* transition, is proportional to the product of the oscillator strength *f*_1*s*→*np*_ and the ground exciton density *n*_1*s*_ (refs 39, 40, 55, 56). Because the 3.1 eV pump excitation creates e–h plasma in the band nesting resonance, an accurate estimation of *n*_1*s*_ requires both theoretical study of intervalley scatterings and the corresponding ultrafast measurements, which is beyond the scope of our ultrafast mid-IR intraexcitonic spectroscopy. In fact, the spectral weight from 1*s*→*np* is not only proportional to the population, but also depends on the probability of finding an empty final *np* state. As discussed about the transient spectra dynamics above, the photoexcited unbound e–h pairs experience rapid relaxation and start to form a ground-state exciton within ∼0.4 ps. It is strictly true that the *np* exciton population is negligible only at Δ*t*⩾0.4 ps. Similar studies on 1D and quasi-2D quantum-well structures have shown that the contribution from *np*→continuum is negligible^33^,^54^,^56^,^57^,^58^. We found that the spectral fit matches well the measured data when we used up to three mid-IR oscillators, with the following transition energy *E*_*n*_ of *E*_1_=0.27 eV, *E*_2_=0.31 eV, and *E*_3_=0.36 eV. On the basis that the observed *E*_*n*_s do not vary Δ*t*, we fix *E*_*n*_ to fit the time-resolved mid-IR spectra, but vary *S*_1*s*→*np*_ and the phenomenological exciton broadening parameter Γ. For the IR transients, the spectra are featureless representing the step-function-like 1*s* to the continuum transition^21^; this featureless IR spectrum is deviated from the step-like absorption at Δ*t*≤0.4 ps, which might be due to the time-dependent thermalization process after the pump excitation.

Given that the magnitude of *S*_1*s*→*np*_ differs only by a factor of 2 for each *E*_*n*_, these transitions cannot simply be assigned to the phenomena taking place within single exciton Rydberg series of the A (or B) exciton branch. This is because even when the nonhydrogenic excitonic nature of a monolayer TMDC is considered, where a strongly (weakly) screened Coulomb potential is dominant when *n*≤2 (*n*⩾3) (ref. 21), the spectral weight should be substantially decreased by nearly an order of magnitude with increasing *n*, which is too large to account for our measurement results. Of course, care should be taken to estimate the precise strength of intraexcitonic transition in a monolayer TMDC because the nonhydrogenic exciton is dominant when *n*≤2, thus the 1*s*→*np* transition can deviate from the hydrogenic excitonic nature for *n*⩾3. This is because any intraexcitonic 1*s*→*np* transition depends both on the wavefunction of the *n*th exciton as well as the 1*s* exciton state, and the latter certainly deviates from the 2D-hydrogen model. Recent PLE^23^ revealed that the energy levels of the exciton Rydberg series are 1.88 eV (1*s*), 2.05 eV (2*s*) and 2.15 eV (3*s*) for A exciton and 2.03 eV (1*s*), 2.24 eV (2*s*) and 2.34 eV (3*s*) for B exciton. By considering 0.15 eV energy splitting between A and B excitons and the difference of reduced exciton masses of 0.25*m*_0_ (A exciton) and 0.28*m*_0_ (B exciton)^18^, we estimated the intraexcitonic transition energies of 0.27 eV for *E*_1A→3A_, 0.31 eV for *E*_1B→3B_ and 0.36 eV for *E*_1A→2B_, which are exactly matched our measured intraexcitonic transition energy *E*_*n*_. Interestingly, these values are somewhat deviated from the GW–Bethe–Salpeter prediction^19^, possibly due to the substrate dielectric screening effect. Nevertheless, our measurements agree well with the experimental PLE investigation due to similar dielectric constant of CaF_2_ and fused silica^23^ as a substrate. Although there is a small difference (∼20 meV) for the A exciton energy between PLE (1.88 eV) and our photocurrent spectra and ultrafast absorption measurement (1.86 eV, Supplementary Notes 1 and 3), the difference is very marginal^19^,^28^,^30^,^31^ and the intraexcitonic spectroscopy can measure the energy difference between 1*s* and *np*, regardless of the A-exciton resonance. In accordance with PLE and our mid-IR measurements, we expect the fundamental 1*s*→2*p* would be 0.17 eV for the A exciton and 0.21 eV for the B exciton, and this is beyond our capability of tuning the mid-IR spectrum. Therefore, as schematically shown in Fig. 3a, we understand our intraexcitonic transition energy of *E*_1_ as 1*s*_,A_→3*p*_,A_ within A exciton, *E*_2_ as 1*s*_,B_→3*p*_,B_ within B exciton and *E*_3_ as 1*s*_,A_→2*p*_,B_ between A and B exciton. Indeed, our energy assignment well-corroborates a recent many-body Bethe–Salpeter prediction on the nonhydrogenic characters of excited excitons^19^,^20^,^28^,^30^,^31^, underscoring a distinct capability of our intraexcitonic spectroscopy in measuring the relative energy difference between 1*s* and *np*. For the exciton broadening parameter Γ, since the effective mass of A and B exciton is different, Γ (=28.2 meV) for 1*s*_,A_→3*p*_,A_, Γ (=37.4 meV) for 1*s*_,B_→3*p*_,B_ and Γ (=30 meV) for 1*s*_,A_→2*p*_,B_ are slightly different due to the different exciton dispersion.

### Dynamics of 1*s*→*np* intraexcitonic spectral weights

For further analysis, we show the temporal dynamics of *S*_1*s*,A→3*p*,A_ (Fig. 3c, blue), *S*_1*s*,B→3*p*,B_ (Fig. 3d, orange) and *S*_1*s*,A→3*p*,A_ (Fig. 3e, green). We identify three different kinetic regimes: immediately after the pump, the rising transients of all three spectral weights show similar behaviours, representing the hot-carrier relaxation from the quasi-continuum to the A and B exciton branch. This kinetics clearly differs from the dynamics of 1.86 eV probe (Fig. 3b), where the latter arises from the quasi-instantaneous bleaching dynamics. At 0.4≤Δ*t*≤0.7 ps, the dynamics of *S*_1*s*,B→3*p*,B_ rapidly decrease, while the peak *S*_1*s*,A→3*p*,A_and *S*_1*s*,A→3*p*,B_ emerge ∼0.3 ps later. Because the 1*s* B exciton is 0.15 eV higher than that of A exciton (Fig. 3a), the 1*s* B exciton serves as a population supplier to the energetically lower 1*s* A exciton, thereby the two transients show a complementary dynamics. At longer Δ*t*>0.7 ps, because the 1*s* A excitons are thermalized and reaches a quasi-equilibrium condition, the dynamics of *S*_1*s*,A→3*p*,A_ nearly follows that of *S*_1*s*,A→2*p*,B_. This highlights that although *S*_1*s*,A→3*p*,A_ and *S*_1*s*,A→3*p*,B_ are spectrally separated apart, that is 0.27 and 0.36 eV, respectively, both transients are closely interrelated because these absorptions originate from the same 1*s*_,A_ ground state exciton.

## Discussion

At an elevated temperature, the free-carrier absorption from 1*s*, 2*s*, 2*p*, 3*s*, 3*p*… may contribute to the increased probe absorption with Δ*T*/*T*_0_<0. This scenario typically shows a strong temperature dependence of the relaxation rate, in which the higher temperature the larger the electron–phonon scattering rate, resulting in the dynamics to be highly temperature dependent. Here given that the formation time scale of the 1*s* exciton is very fast within 0.4 ps (see Figs 2 and 3) and the Drude scattering rate cannot be extended to the mid-IR range (Supplementary Note 2), the contribution of *np*→continuum transition may be very insignificant to the temperature-dependent mid-IR intraexcitonic response. Figure 4a shows that our mid-IR transients, fitted by a biexponential function, exhibit nearly temperature independent of the relaxation components (Fig. 4b,c). This implies that the effect of free-carrier absorption is negligible. We additionally show in Fig. 4b that the recombination of excitons arises on sub-ps and tens of ps time scale. At *T*=77 K, the mid-IR peak |Δ*T*/*T*_0_| linearly increases with *F* up to 32.5 μJ cm^−2^ (equivalent to e–h pair density of 9.86 × 10^12^ cm^−2^) (refs 8, 41). The linear *F*-dependence reflects that there exists no high-order nonlinear excitonic interaction, ensuring that our mid-IR transients represent the first-order population dynamics. A recent below-gap-probe study^45^ reported very similar relaxation times to our results. These time components were explained using defect-assisted exciton recombination. Given that we observed negligible *F*-dependent relaxation dynamics (Fig. 4e,f), we can infer that our mid-IR decay transients do not arise from the photoinduced absorption of filled e–h pair in the localized states, but arise from the exciton capture into the defects.

In summary, we report the experimental observation of the 1*s* intraexcitonic transition. Recently, Poellmann *et al*.^47^ investigated a similar investigation of intraexcitonic transition in monolayer WSe_2_, reporting the presence of strong absorption in a 2D TMDC, whose fundamental optical absorption originates from the 1*s* ground exciton. Our ultrafast mid-IR measurements reveal twofold 1*s*→3*p* transition energies to be 0.27 eV and 0.31 eV for A and B exciton, respectively. We additionally uncover an intraexcitonic relaxation channel of 1*s*→2*p* to be 0.36 eV between 1*s* A and 2*p* B exciton. The large exciton-binding energy due to the non-local dielectric screening ensures not only 1*s*→2*p* transition^47^ to be observable, but also a higher-order transition of 1*s*→3*p* in a monolayer 2D TMDC at an elevated temperature, which cannot be accessible using conventional interband spectroscopy, or any in quasi-2D quantum-well structures. In addition, looking to the future, the availability of electric-gate tuning may enable to investigate the coherent many-body inter-excitonic correlations among exciton, biexciton^38^,^59^ and trion^12^,^13^,^18^,^48^ in a time-resolved controlled manner, which is non-trivial to study in other low-dimensional inorganic semiconductor structures.

## Methods

### Ultrafast optical pump–probe spectroscopy

Using 250 kHz, 50 fs Ti/sapphire laser system (Coherent RegA 9050), optical parametric amplifier (Coherent OPA 9850) yields signal (0.77–1.12 eV) and idler (0.47 eV–0.67 eV) pulses that are used to generate mid-IR pulse (0.23–0.37 eV) via difference frequency generator (Coherent DFG). The idler and DFG output serve as the probe pulse in the IR and mid-IR range, respectively. The chirp of mid-IR pulse is discussed in Supplementary Note 5. High-energy interband response was measured by using a white-light continuum (1.76–2.03 eV) generated by focusing 1.55 eV pulses into a 1 mm sapphire disk. For the group-delay dispersion (GDD) of the white-light continuum pulse, we compensated using a pair of prism, and further checked the GDD-induced delay via cross-correlation of the white-light pulse and 1.55 eV pulse, whose details are explained in the Supplementary Note 3. The 3.1 eV pump pulse was created by second harmonic generation of 1.55 eV pulse in a 1-mm-thick beta barium borate (BBO) crystal. Due to the combination of OPA and DFG, where both signal and idler from OPA were used to generate the mid-IR DFG output, the only available seed pulse for 3.1 eV pump pulse was 1.55 eV in our system, so that the mid-IR measurement with resonant pump excitation at either A or B 1*s* exciton was not possible in our current system. For the each mid-IR or IR measurement, pump pulse and probe pulse are simultaneously focused on the sample in the cryostat equipped with two CaF_2_ windows, and pump–probe delay is controlled by a mechanical delay stage (Newport M-IMS300LM). The spot size of our pump and probe beams were 100 μm, and 50 μm, respectively, which were simultaneously focused using *f*=50 mm lens before the temperature-controlled vacuum cryostat. In our optical geometry, the 3.1 eV pump passes through a mechanical delay stage, so called ‘pump delay'. Because the pump delay is recorded in a computer as an absolute length, we performed cross-correlation measurement to estimate the probe delay using BBO (visible upconversion) and KTA (mid-IR upconversion) crystals. More detailed information for determining the pump–probe ‘time-zero' is explained in Supplementary Note 6. Differential transmission signal (Δ*T*/*T*_0_) was recorded in a lock-in amplifier (Stanford Research Systems SR850) with 10 kHz chopping frequency (Scitec 300CD). The schematics of mid-IR and IR setup are illustrated in the Supplementary Fig. 9.

## Additional information

**How to cite this article:** Cha, S. *et al*. 1*s* intraexcitonic dynamics in monolayer MoS_2_ probed by ultrafast mid-infrared spectroscopy. *Nat. Commun.* 7:10768 doi: 10.1038/ncomms10768 (2016).

## Supplementary Material

Supplementary Information Supplementary Figures 1-10, Supplementary Notes 1-8 and Supplementary References.
