# Supplementary material for: 1s-intraexcitonic dynamics in monolayer MoS2 probed by ultrafast mid-infrared spectroscopy
Source: Nat Commun. 2016 Feb 25;7:10768. doi: 10.1038/ncomms10768 (PMC4773417; doi:10.1038/ncomms10768)
Supplement: Supplementary Information — Supplementary Figures 1-10, Supplementary Notes 1-8 and Supplementary References. [file ncomms10768-s1.pdf]

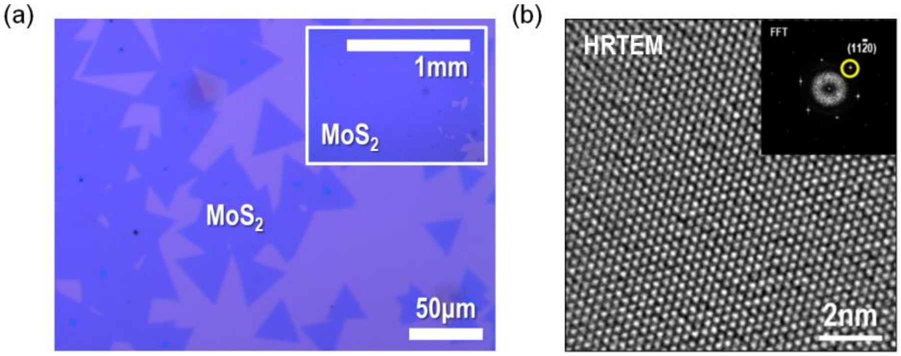


**Supplementary Figure 1 | Optical microscope image of large area $MoS_2$ monolayers and in-plane transmission electron microscopy (TEM) image. (a)** Optical microscope image of polycrystalline $MoS_2$. (**b)** The TEM image of $MoS_2$ monolayer crystals.

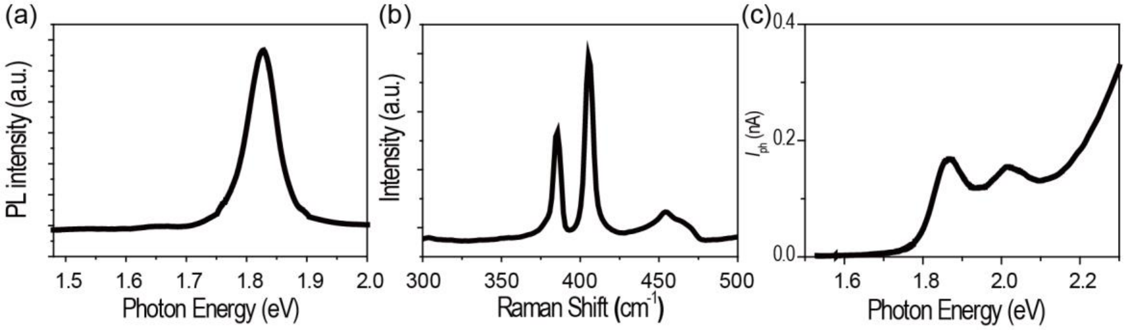


**Supplementary Figure 2 | Optical characteristics of monolayer $MoS_2$ (a)** PL spectrum of monolayer $MoS_2$ with excitation photon energy of 2.33 eV. **(b)** Raman scattering spectrum of $MoS_2$. Two pronounced phonon modes indicate the monolayer character of $MoS_2$. **(c)** Photocurrent spectrum was obtained for $MoS_2$ monolayer in the photon energy range from 1.5 to 2.3 eV.

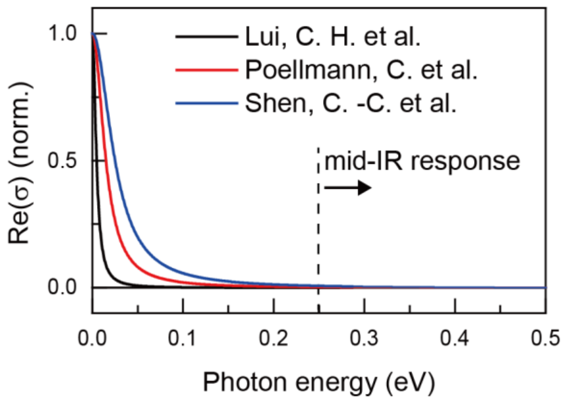


**Supplementary Figure 3 | Calculated real part of Drude conductivity of monolayer $MoS_2$.** The scattering rates are 7.2 THz[1], 23 THz[2], and 38.4 THz[3]. The dashed line indicates the lower limit of our mid-IR spectra.

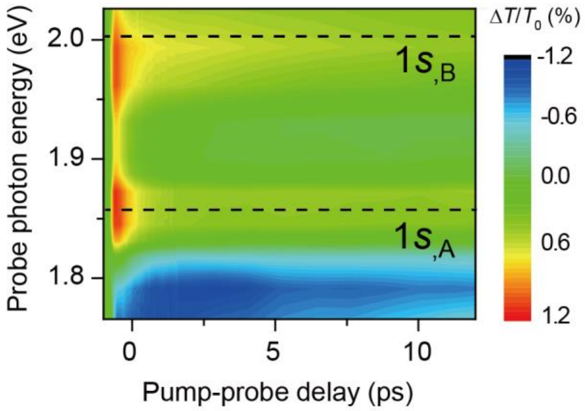


**Supplementary Figure 4 | Temporally- and spectrally-resolved dynamics over interband transition energy range.** Transient $\Delta T/T_0$ spectra are obtained using ultrafast white-light continuum spectroscopy. The GDD-induced delay is compensated as discussed in Supplementary Note 3.

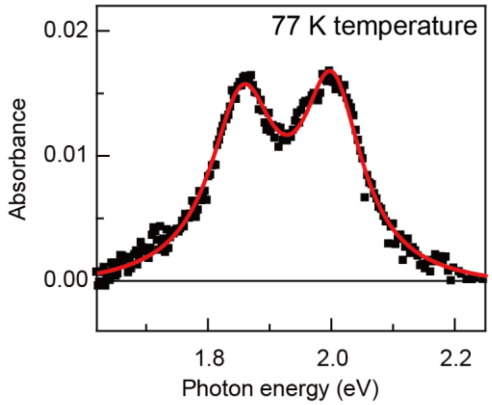


**Supplementary Figure 5 | Equilibrium absorption response of monolayer $MoS_2$.** The measurement was performed at temperature of 77 K. The data (black squares) are fitted by Supplementary Eq. 2 (red line).

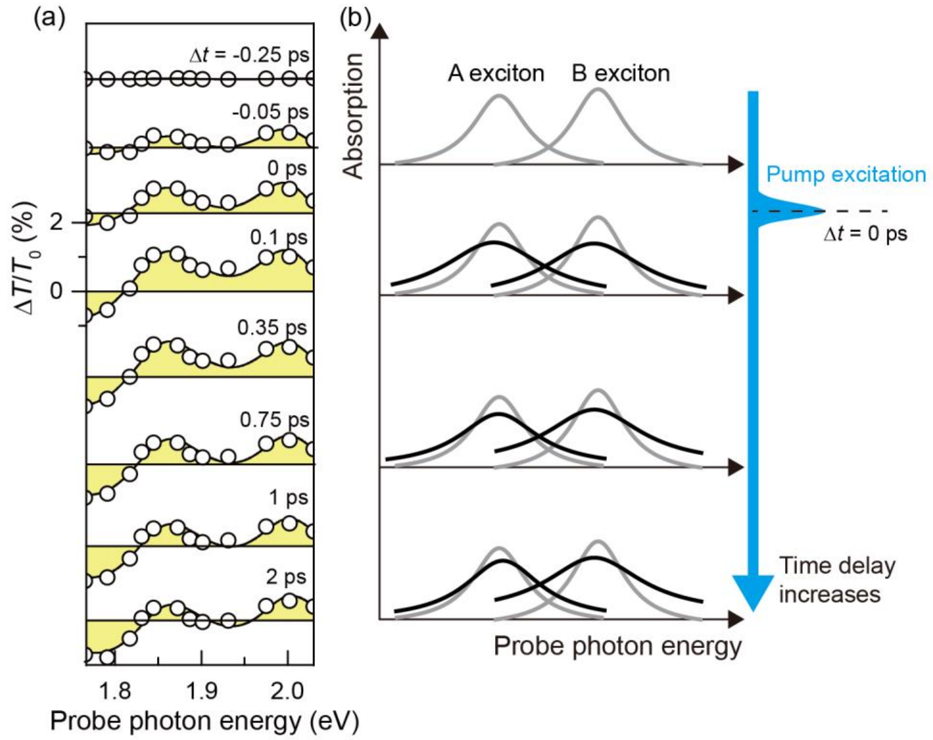


**Supplementary Figure 6 | Details of interband dynamics. (a)** Temporally- and spectrally-resolved (black open circles) dynamics for several pump-probe delays are shown. **(b)** Schematic illustrations of the time-dependent exciton dynamics. Gray is the equilibrium and black solid is the nonequilibrium.

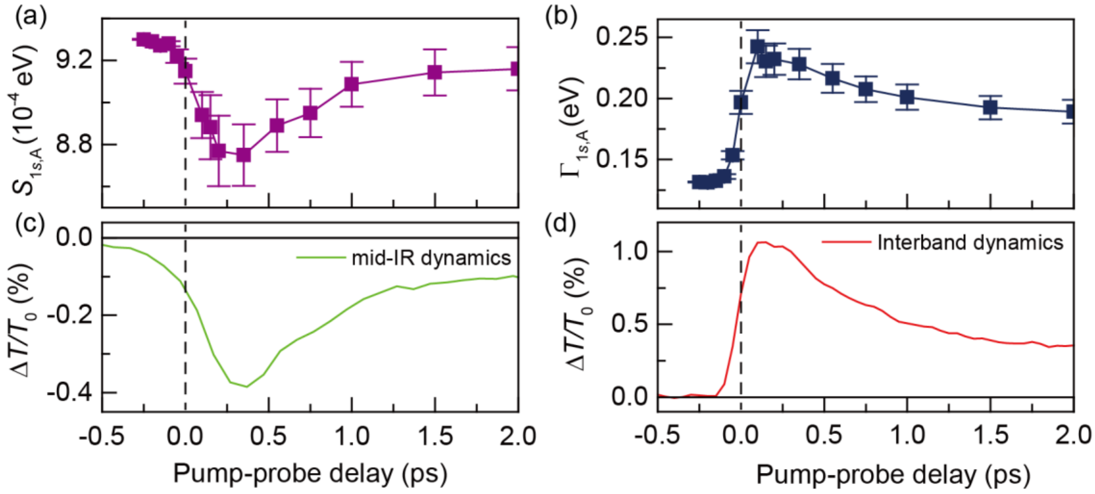


**Supplementary Figure 7 | Comparison between transient response shown in Fig. 1b and extracted fit parameters. (a,b)** Transient spectral weight $S_{1s,\mathrm{A}}$ and linewidth broadening $\Gamma_{1s,\mathrm{A}}$ of A exciton obtained from the interband $\Delta T/T_0$ are shown in (a) and (b), respectively. **(c,d)** For comparison, we show the mid-IR and interband dynamics in (c) and (d), respectively.

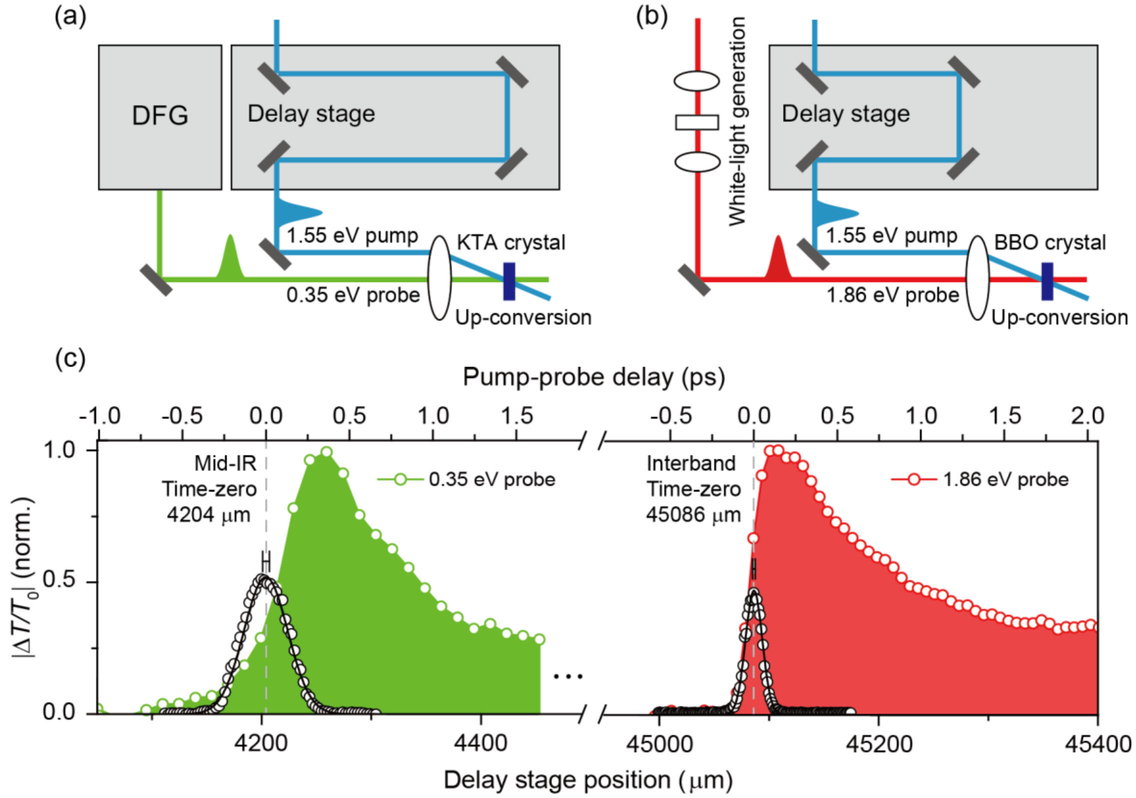


**Supplementary Figure 8 | Illustration of the synchronization process. (a,b)** Experimental setup for cross-correlation of the mid-IR (a) and the visible probe (b). **(c)** The synchronization of cross-correlation and transient dynamics for the mid-IR (left) and the visible probe (right) is shown. The full-width-at-half-maximum (FWHM) is 320 fs for the mid-IR pulse, and 100 fs for the visible pulse. For each cross-correlation, error bar of each time-zero is displayed above the correlation data.

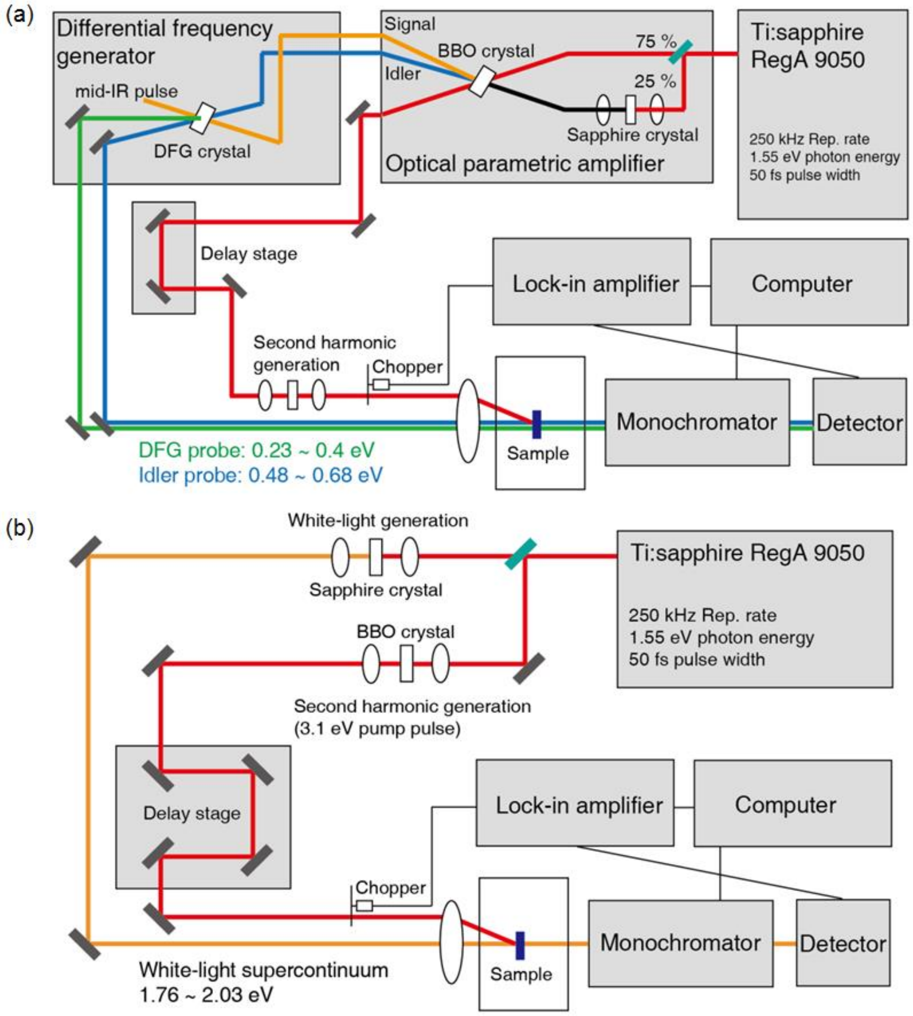


**Supplementary Figure 9 | Experimental setup for ultrafast optical pump-probe spectroscopy. (a)** Ultrafast 3.1 eV pump and mid-IR/IR probe spectroscopy. **(b)** Ultrafast white-light continuum spectroscopy. Experimental details are described at the method section in the main manuscript.

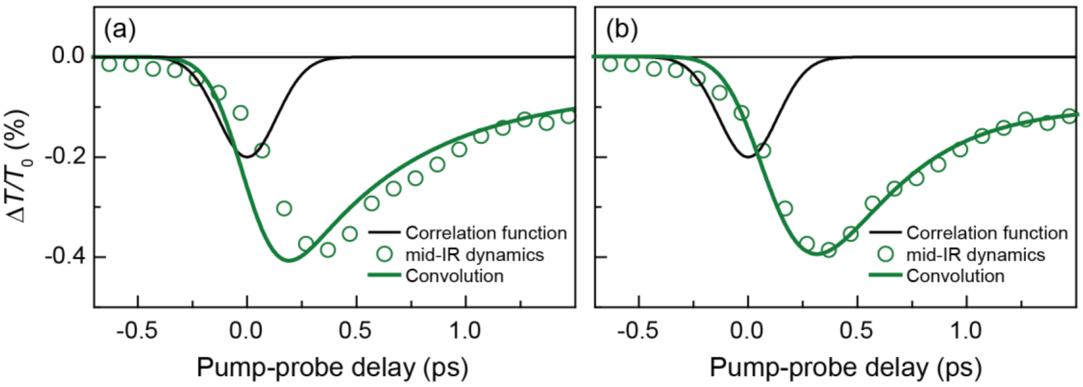


**Supplementary Figure 10 | Convolution fit of mid-IR dynamics. (a)** Convolution with immediate response at rising edge. **(b)** Convolution with exponential rising lifetime of 0.4 ps. Measured mid-IR dynamics shown in Fig. 1b (green dots), cross-correlation (black line), and the convolution fit (green solid) are shown.

**Supplementary Note 1. Preparation of CVD-grown monolayer $MoS_2$**

Vapor-phase grown $MoS_2$ monolayer crystals of tens of $\mu$m in the lateral size are synthesized on $SiO_2$/Si substrate from solid $MoO_3$ powder precursors with S powders in $N_2/H_2$ gas flow inside a 12-in. hot-wall quartz-tube at 660 °C[4,5]. A typical lateral size of a single crystal (a triangular shape) is 20-30 μm, which shows the mm-size scale of the merged single-crystal individual triangular flake. Each flake is randomly oriented with each other with grain boundaries, resulting in a polycrystalline nature of the large-scale $MoS_2$ sheet. The optical microscope image of $MoS_2$ is also shown in Supplementary Figure 1a. For the characterization of monolayer $MoS_2$, the atomic structure of $MoS_2$ monolayer crystal was investigated by high-resolution in-plane transmission electron microscopy (TEM), as shown in Supplementary Figure 1b, whose upper inset is the corresponding fast Fourier transformation pattern which shows clear hexagon.

In addition, PL spectrum was obtained for $MoS_2$ monolayer in the photon energy range from 1.5 to 2.0 eV with excitation photon energy of 2.33 eV. $MoS_2$ monolayer exhibits the PL peak at the interband A-exciton energy (1.83eV) as displayed in Supplementary Figure 2a. Note that the PL emission is red-shifted compared to the photocurrent absorption spectrum (Supplementary Figure 2c) due to the Stokes shift. Supplementary Figure 2b displays Raman scattering spectrum, which shows two distinct Raman peaks which corresponds to $E_{2g}$ (386cm$^{-1}$) and $A_{1g}$(405cm$^{-1}$) vibration modes of the $MoS_2$ monolayer. The difference of these two modes (19cm$^{-1}$) indicates its monolayer character. Finally, photocurrent spectrum was obtained for $MoS_2$ monolayer in the photon energy range from 1.5 to 2.3 eV. This measurement is equivalent to the optical absorption to some certain extent. $MoS_2$ monolayer exhibits an abrupt increase near 1.8 eV and two pronounced A (1.86 eV) and B (2.01 eV) exciton peaks.

**Supplementary Note 2. Drude analysis for temperature-dependent free-carrier absorption**

First of all, if our mid-IR measurements represent the n*p*→continuum transition, it does not necessary show temperature dependence. Although the nonhydrogenic nature of excitons is strictly hold for $n$=1,2 in a monolayer TMDC, Fig. 1b in the manuscript shows that the formation time scale of the 1*s* exciton is very fast within 0.4 ps. We discussed that the photoexcited unbound *e*-*h* pair experiences rapid relaxation and forms a ground-state exciton within approximately 0.4 ps. Based on the convolution fit of our mid-IR transients, the spectrally measured Fig. 2 data consolidated that it is strictly true that the n*p* exciton population is negligible at $\Delta t \geq 0.4$ ps. The detailed procedure for the convolution is discussed in Supplementary Note 8. Based on these analyses, we believe that the above-gap excitation supplies a very marginal population in the intermediate n*p* states, $\Delta t \geq 0.4$ ps after the pump. Similar studies (although they are not done on an ideal 2D material) have shown that the contribution from n*p*→continuum is negligible for an ultra-thin Si[6], 1D carbon nanotube[7], and quasi-2D quantum-well structures[8,9].

For the thermalized carriers into the band edge, the spectral response is presumably dominated by the temperature-dependent Drude response. We understand that if our mid-IR signal is originated from the thermalized carriers, the response will be governed by the high-energy tail of the Drude response, as a consequence of the intraband transition[10,11]. This high-energy tail of Drude response significantly depends on the scattering rates, among which the dominant temperature-dependent contribution is supposed to be the electron-phonon scattering. We thoroughly searched for the existing studies, especially ultrafast THz spectroscopic

investigations on a monolayer TMDC, and found that there are three representative results[1-3]. We extracted the Drude width, and plotted the simulation results based on the parameters. The results are shown in Supplementary Figure 3. Obviously, we see that the Drude response cannot be extended out all the way to the mid-IR frequency even if we used a highest value of a Drude scattering width.

**Supplementary Note 3. Compensation of GDD-induced time delay and ultrafast visible dynamics of $MoS_2$**

In the case of white-light continuum source, we performed a cross-correlation measurement between the broadband white-light and the 1.55 eV pulse in BBO crystal[12,13]. Indeed, the white-light continuum has a lot of group-delay dispersion (GDD), which can be expressed as the following equation

$$\tau_{group}(\omega) = \frac{\partial \phi}{\partial \omega}\Big|_{\omega_0} + \frac{\partial^2 \phi}{\partial \omega^2}\Big|_{\omega_0} (\omega - \omega_0) + \frac{1}{2!}\frac{\partial^3 \phi}{\partial \omega^3}\Big|_{\omega_0} (\omega - \omega_0)^2 + \cdots, \qquad (1)$$

where $\phi$ is the pulse phase, and $\omega_0$ is the center frequency. The second and third order terms of this equation are main factors for the GDD. Using the cross-correlation technique, we can experimentally determine the GDD-induced delay, and compensate the broad spectrum onto an absolute time-zero. The extracted GDD-induced delay is then used to correct the time-zero for each probe energy, and the results are included for the two-dimensional plot of transient $\Delta T/T_0$ response. Supplementary Figure 4 displays corresponding spectrally-resolved dynamics of visible response. Pronounced peaks at 1.86 eV and 2.01 eV indicate the 1*s* ground A and B exciton, respectively (see dashed lines). These A and B peaks are well consistent with the photocurrent spectrum shown in Supplementary Figure 2c. The rapid rising dynamics were

observed for all spectral range, corroborating the quasi-instantaneous ground-state bleaching induced by free carriers, as discussed in the manuscript.

**Supplementary Note 4. Analysis of the interband spectroscopy results**

For the quantitative analysis of visible dynamics shown in Supplementary Figure 4, we first performed measurements of equilibrium absorption. The absorbance $\alpha(\omega)$ was obtained by measuring the transmittance contrast ($\alpha = -\log(T_{\mathrm{sample}}/T_{\mathrm{substrate}})$) of the sample in vacuum at 77 K, and the result is shown in Supplementary Figure 5; here, the backscattered signal was eliminated by a polynomial fit to the measured data.

The pronounced peak centered at 1.86 eV, and 2.01 eV is the $1s_{,\mathrm{A}}$, and $1s_{,\mathrm{B}}$ resonances, respectively. The resonance energy, absorption spectral weight, and broadening are obtained via a fit to the data using the following function:

$$\alpha(E) = \sum_{A,B} \frac{\frac{1}{2} S_{\mathrm{i}} \Gamma_{\mathrm{i}}}{(E - E_{\mathrm{i}})^2 + (\frac{1}{2}\Gamma_{\mathrm{i}})^2}, \qquad (2)$$

where $E_{\mathrm{i}}$, $S_{\mathrm{i}}$, and $\Gamma_{\mathrm{i}}$ are resonance energy, spectral weight, and exciton linewidth broadening, respectively. Having obtained the equilibrium absorption spectrum, we show the time-dependent spectral dynamics shown in Supplementary Figure 6a. For each pump-probe delay, the differential transmission ($\Delta T/T_0$) spectrum was fitted by subtracting the equilibrium transmission from the nonequilibrium response. For the conversion from $\Delta\alpha/\alpha_0$ to $\Delta T/T_0$, we follow below Beer's law,

$$\Delta\alpha = \alpha_{\text{neq.}} - \alpha_{\text{eq.}} = -\log\left(1 + \frac{\Delta T}{T_0}\right), \tag{3}$$

where $\alpha_{\text{eq.}}$ and $\alpha_{\text{neq.}}$ are absorbance in equilibrium and nonequilibrium, respectively[14]. In Fig. S6b, we show the corresponding schematics of the time-dependent A and B exciton resonances.

Time-dependent changes of $S_{1s,\mathrm{A}}$ and $\Gamma_{1s,\mathrm{A}}$ obtained from the above interband spectroscopy are shown in Supplementary Figure 7. Notably, we see that $\Gamma_{1s,\mathrm{A}}$ closely follows the transient of interband A exciton resonance (Supplementary Figure 7b and 7d). The photoexcitation into the quasi-continuum of unbound states generates a significant amount of free-carriers, and the exciton linewidth broadening is well described by the exciton-free carrier scattering. As demonstrated in earlier studies on quantum-well structures[14-16], the presence of the free *e*-*h* plasma and the corresponding exciton-free carrier scattering is not unexpected. For the population dynamics of A excitons, the photoinduced spectral weight $S_{1s,\mathrm{A}}$ shows a rather slowly decay feature, as shown in Supplementary Figure 7a and 7c. Interestingly, the initial dynamics strongly resembles the mid-IR transient. We additionally note that the relative changes of $S_{1s,\mathrm{A}}$ is less than 6 % ($5.5\times10^{-5}$ / $9.3\times10^{-4}$ = 0.059). On the contrary, the corresponding changes of $\Gamma_{1s,\mathrm{A}}$ is much larger with almost 80 % (0.131 meV / 0.242 meV = 1.84), supporting our rationale that the contribution of exciton linewidth broadening is much larger than the population contribution to the measured interband dynamics.

### Supplementary Note 5. Characterization of ultrashort mid-IR pulse

The imposed chirp of the mid-IR pulse would affect not only the rising transient (Fig. 1b and Fig. 3), but also influence the spectrally-resolved mid-IR transient (Fig. 2, left panel of mid-IR data).

To determine the effect of mid-IR chirp, we carefully measured the wavelength-resolved cross-correlation of the mid-IR and 1.55 eV (800 nm) pulse. The output of amplifier 1.55 eV (800 nm) pulse is recompressed to ~ 50 fs with an SF-10 prism pair to compensate any accumulated dispersion through the optics. The upconversion was performed though the same KTA nonlinear crystal with a GaAsP PMT (Hamamatsu H7421-40) with a grating spectrometer equipped with SRS400 photon-counting electronics[17]. By measuring the generated sum-frequency signal as a function of the wavelength, the spectral characteristics of mid-IR pulse were obtained. The mid-IR pulse is imposed by a very small negative chirp $C_{\text{chirp}} < 0$, but the wavelength-dependent temporal delay is only 60 fs from 0.25 eV to 0.37 eV.

**Supplementary Note 6. Time-zero determination**

It is important to note that the two experiments (visible and mid-IR probes) require sharing a common origin to exactly determine the time-zero of the pump-probe delay (Fig. 1b). In our optical geometry, the 3.1 eV pump (frequency doubling of 1.55 eV from BBO) passes through a mechanical delay stage, so called “pump delay”. Because the pump delay is recorded in a computer as an absolute length, a remaining issue is how to exactly measure the probe delay. Here, we mounted BBO (visible upconversion) and KTA (mid-IR upconversion) right next to each other, such that we made our best effects to minimize the time-zero error between the two experiments. In the following, we discussed how we performed again the up-conversion experiments for both experiments, and how we carefully calibrated the “time-zero”.

To calibrate the exact “time-zero”, we performed two separated up-conversion experiments. During this process, the optical geometry is set to exactly same as the pump-probe measurement as shown in Fig. 1b, including dispersion in 3 mm-thick $CaF_2$ cryostat window. The experiment

setup is depicted in Supplementary Figure 8. By measuring cross-correlation signal of each probe pulse, the absolute delay position of the pump delay for visible and mid-IR probe measurements are recorded, and FWHM of each probe pulse has been measured (see black line in Supplementary Figure 8c). Considering this absolute delay position, we have plotted the visible and mid-IR probe dynamics simultaneously, as shown in Fig. 1b. The time difference of the rising peaks between mid-IR dynamics and 1.86 eV probe has been observed about 0.2 ps.

**Supplementary Note 7. Experimental setup of ultrafast pump-probe spectroscopy**

As discussed on method in our manuscript, 50 fs Ti:sapphire laser system (Coherent RegA 9050) has been employed to perform ultrafast pump-probe spectroscopy. Experimental setups of two distinct measurements are illustrated in Supplementary Figure 9. As shown in Fig. 9a, optical parametric amplifier (Coherent OPA 9850) yields signal (0.77 ~ 1.12 eV) and idler (0.47 eV ~ 0.67 eV) pulses that are used to generate mid-IR pulse (0.23 ~ 0.37 eV) via difference frequency generator (Coherent DFG). High-energy interband response was measured by using a white-light continuum (1.76 ~ 2.03 eV) generated by focusing 1.55 eV pulses into a 1 mm sapphire disk (Supplementary Figure 9b).

**Supplementary Note 8. Convolution fit of mid-IR transients**

We can further corroborate that the relatively slow rising mid-IR transient (Fig. 2) is consistent with the intraexcitonic relaxation and the corresponding 1*s* exciton formation dynamics (Fig. 3). In fact, the ground exciton formation in atomically thin 2D materials has been previously investigated in van der Waals heterostructures[18]. The procedure is as following. The measured

mid-IR 1*s* exciton signal can be accounted for by a convolution of the mid-IR cross-correlation function and the exciton response function. If the 1*s* exciton response is instantaneous—that means, the mid-IR and band-to-band response occurs from the same origin, then the measured mid-IR dynamics should be matched with a convolution result using the cross-correlation and the instantaneous response. Supplementary Figure 10a clearly shows that this assumption fails to explain the measured data (empty circles). A more natural way of explanation is that the photoexcited unbound *e*-*h* pairs should take a certain amount of time before the bound *e*-*h* pair formation occurs. Spectrally resolved data of Fig. 3 in the revised manuscript show that it takes approximately 0.4 ps for the exciton formation. We included this ~ 0.4 ps delay kinetics in the exciton response function $\Theta(t)$ as following;

$$\Theta(t) = \Phi(t)\left(1 - e^{-\frac{t}{\tau_{\text{rise}}}}\right)\left(A_1 e^{-\frac{t}{\tau_1}} + B_1 e^{-\frac{t}{\tau_2}}\right), \qquad (4)$$

where $\Phi(t)$ and $\tau_{\text{rise}}$ are a unit step function and a rising time constant, respectively. The third term of $\Theta(t)$ shows bi-exponential decay dynamics. Then, we see that in Supplementary Figure 10b the measured data can be reproduced well after this formation kinetics is incorporated in the convolution.

**Supplementary References**


1 Lui, C. H. *et al*. Trion-Induced Negative Photoconductivity in Monolayer $MoS_2$. *Phys*. *Rev*. *Lett*. **113**, 166801 (2014).

2 Poellmann, C. *et al*. Resonant internal quantum transitions and femtosecond radiative decay of excitons in monolayer $WSe_2$. *Nat*. *Mater*. **14**, 889-893 (2015).

3 Shen, C. -C. *et al*. Charge dynamics and electronic structures of monolayer $MoS_2$ films grown by chemical vapor deposition. *Appl*. *Phys*. *Exp*. **6**, 125801 (2013).

4 Najmaei, S. *et al*. Vapour phase growth and grain boundary structure of molybdenum disulphide atomic layers. *Nat. Mater*. **12**, 754-759 (2013).

5 Heo, H. *et al*. Rotation-misfit-free heteroepitaxial stacking and stitching growth of hexagonal transition-metal dichalcogenide monolayers by nucleation kinetics controls. *Adv. Mater*. **27**, 3803-3810 (2015).

6 Suzuki, T. & Shimano, R. Time-resolved formation of excitons and electron-hole droplets in Si studied using terahertz spectroscopy. *Phys*. *Rev*. *Lett*. **103**, 057401 (2009).

7 Wang, J., Graham, M. W., Ma, Y., Fleming, G. R. & Kaindl, R. A. Ultrafast spectroscopy of midinfrared internal exciton transitions in separated single-walled carbon nanotubes. *Phys. Rev, Lett*. **104**, 177401 (2010).

8 Kaindl, R. A., Hägele, D., Carnahan, M. A. & Chemla, D. S. Transient terahertz spectroscopy of excitons and unbound carriers in quasi-two-dimensional electron-hole gases. *Phys*. *Rev*. *B* **79**, 045320 (2009).

9 Kaindl, R. A., Carnahan, M. A., Hägele, D., Lövenich, R. & Chemla, D. S. Ultrafast terahertz probes of transient conducting and insulating phases in an electron–hole gas. *Nature* **423**, 734-738 (2003).

10 Ren, L. *et al*. Terahertz and infrared spectroscopy of gated large-area graphene. *Nano Lett*. **12**, 3711-3715 (2012).

11 Wagner, M. *et al*. Ultrafast and nanoscale plasmonic phenomena in exfoliated graphene revealed by infrared pump–probe nanoscopy. *Nano Lett*. **14**, 894-900 (2014).

12 Urayama, J. Ph.D. thesis, The University of Michigan (2002).

13 Maciejewski, A. *et al*. Transient absorption experimental set-up with femtosecond time resolution. Femto- and picosecond study of DCM molecule in cyclohexane and methanol solution. *Mol. Struct*. **555**, 1 (2000).

14 Wake, D. R., Yoon, H. W., Wolfe, J. P. & Morkoç, H. Response of excitonic absorption spectra to photoexcited carriers in GaAs quantum wells. *Phys*. *Rev*. *B* **46**, 13452-13460 (1992).

15 Knox, W. H. *et al*. Femtosecond excitation of nonthermal carrier populations in GaAs quantum wells. *Phys*. *Rev*. *Lett*. **56**, 1191-1193 (1986).

16 Knox, W. H., Chemla, D. S., Livescu, G., Cunningham, J. E. & Henry, J. E. Femtosecond carrier thermalization in dense Fermi seas. *Phys*. *Rev*. *Lett*. **61**, 1290-1293 (1988).

17 Choi, H. *et al*. Ultrafast Rabi flopping and coherent pulse propagation in a quantum cascade laser. *Nat. Photon*. **4**, 706-710 (2010).

18 Hong, X. *et al*. Ultrafast charge transfer in atomically thin $MoS_2/WS_2$ heterostructures. *Nat. Nano*. **9**, 682-686 (2014).
